# New Discovery of Curcumin Combination Therapy and Action Mechanism

**DOI:** 10.1155/2020/4793058

**Published:** 2020-03-10

**Authors:** Chongshan Dai, Xiuying Zhang, Keyu Zhang

**Affiliations:** ^1^Department of Surgery, University of Texas Southwestern Medical Center, Harry Hines Blvd, Dallas, TX 5323, USA; ^2^Department of Basic Veterinary Science, College of Veterinary Medicine, Northeast Agricultural University, Harbin, Heilongjiang 150030, China; ^3^Key Laboratory of Veterinary Chemical Drugs and Pharmaceutics, Ministry of Agriculture, Shanghai Veterinary Research Institute, Chinese Academy of Agricultural Sciences, No. 518 Ziyue Road, Minhang District, Shanghai 200241, China

Curcumin (diferuloylmethane, a yellow pigment in spice turmeric) is a natural product polyphenol extracted from the rhizome of *Curcuma longa*. Curcumin has been shown to elicit a strong antioxidant activity by directly scavenging free radicals, even more effectively than vitamin E. It has been widely used in pharmaceutical and medical applications based on the broad spectrum of biological actions, including antibacterial, antiviral, antifungal, and anti-inflammatory activities. Direct curcumin targets include cyclooxygenase 2 (COX-2), lipoxygenase, nuclear factor erythroid 2-related factor 2, toll-like receptor (TLR) 4, transforming growth factor-beta (TGF-*β*)/smad signaling pathway, focal adhesion kinase, glutathione, glycogen synthase kinase- (GSK-) 3 *β*, phosphorylase-3 kinase, xanthine oxidase, pp60 src tyrosine kinase, and ubiquitin isopeptidase, which play important roles in oxidative stress, inflammation, autophagy, ferroptosis, and apoptosis. Therefore, curcumin has been proposed to increase the therapeutic efficiency of some drugs, albeit the potential molecular mechanisms remain unclear. Importantly, available preclinical and phase I/II data suggest that curcumin is well tolerated and has a good safety profile. A classic example is the combination between curcumin and polymyxins whereby in addition to its synergistic antimicrobial activity, curcumin could potentially ameliorate polymyxin-induced unwanted neurotoxicity. With the proposal of concept on precision medicine in the clinic, the underlying molecular mechanism and potential target for the combination therapy are much emphasized.

In this special issue, investigators contribute original research articles and review articles that would facilitate the understanding of the basic mechanisms of curcumin as well as the development of new and promising complementary and alternative strategies for curcumin combination.

Wang et al. reported the neuroprotective effect of curcumin against oxygen-glucose deprivation/reoxygenation (OGD/R) injury in HT22 neuronal cells. They suggested that curcumin can protect OGD/R-induced neuronal apoptotic cell death by inhibiting intracellular ROS accumulation and mitochondria dysfunction. Knockdown of SOD2 by RNA interference (RNAi) attenuated the protective effect of curcumin on OGD/R-induced neuronal cell death, suggesting that SOD2 may be a target of curcumin death.

Wang and Chen reviewed the bidirectional action of curcumin and curcuminoids as well as synthetic curcumin analog on angiogenesis based on the current research findings. They review paper summarized the antiangiogenesis effect of curcumin. Curcumin could regulate multiple factors, including proangiogenesis factor vascular endothelia growth factor (VEGF), matrix metalloproteinase (MMPs), and fibroblast growth factors (FGF), both in vivo and in vitro, and promote angiogenesis under certain circumstances via these factors.

The relationship between gut microbiota and human diseases has been a major topic of interest for many studies. Pan and his colleagues reviewed the effects of gut microbiota in the pharmacological effect of natural products including ginseng, bebeerine, and curcumin. Natural products may cause changes in the composition of intestinal microbiota, microbial metabolites, intestinal tight junction structure, and mucosal immunology. On the contrary, these changes will eventually result in the exertion of the pharmacological effects by treatment with these natural products. This is a new field for the investigation of curcumin's pharmacological effects, and the potential molecular mechanism needs further investigation.

Curcumin has been widely used as a supplementary agent against drug, carbon tetrachloride (CCl_4_), and ethanol-induced liver injury. Inhibiting the TGF-*β* pathway usually was considered as a potential target of curcumin. Zhang et al.'s results implied that a variant of rs17687727 in TGF-*β* receptor-associated protein 1 (TGFBRAP1) (a member of TGF-*β* transmembrane receptors) may influence the susceptibility to antituberculosis drug-induced liver injury in first-line antituberculosis combination treatment in the Han Chinese population in a dependent manner. This result provides the information for potential candidate treatment drugs including curcumin.

Natural products are an important source of drug research for the future. A novel application of natural substances is combination therapy, which consists of the administration of two or more substances, such as conventional chemotherapeutics plus a natural compound or multiple natural compounds at a time. Investigation of potential targets is urgent toward the understanding of molecular mechanisms and the development of key new formulations.

Gong et al. reported an ancient herbal mixture, named Danggui Buxue Tang (DBT_1155_), which comprises four herbs (e.g., *Angelicae Sinensis Radix*, *Astragali Radix, Jujuba Fructus*, and *Zingiberis Rhizoma Recens* in a weight ratio of 36 : 30 : 15 : 20), on the effects of preventing obesity in an in vitro model by using mouse 3T3-L1 fibroblast cells. Their results showed that DBT_1155_ could actuate brown fat-specific gene activations, such as peroxisome proliferator-activated receptor (PPAR*γ*), mitochondrial uncoupling protein 1 (UCP1), and peroxisome proliferator-activated receptor *γ* coactivator 1 (PCG1α). Meanwhile, DBT1155 could decrease lipid accumulation by triggering AMPK signaling.

Tian et al. reported *Cynomorium songaricum* extract exhibited potential therapeutic effect on neuroprotection of ovariectomized rats, and its effect was possibly exerted by p-cAMP-response element binding protein (CREB)/brain-derived neurotrophic factor- (BDNF-) mediated down-regulation of extracellular regulated protein kinases (ERK)/p38 mitogen-activated protein kinase (p38MAPK).

We hope that this special issue can be really special for scientists studying the pharmacological effects of curcumin and discoveries of curcumin combination therapy.

